# Antibacterial Activity of Antibiotic-Releasing Polydopamine-Coated Nephrite Composites for Application in Drug-Eluting Contact Lens

**DOI:** 10.3390/ma15144823

**Published:** 2022-07-11

**Authors:** Min-Seung Kang, Kyung-Jin Moon, Ji-Eun Lee, Young-IL Jeong

**Affiliations:** 1 Department of Ophthalmology, Pusan National University Yangsan Hospital, Yangsan 50612, Gyeongnam, Korea; kangminseung91@gmail.com; 2 School of Medicine, Pusan National University, Yangsan 50612, Gyeongnam, Korea; 3 Research Institute for Convergence of Biomedical Science and Technology, Pusan National University Yangsan Hospital, Yangsan 50612, Gyeongnam, Korea; 4 Research Center, Dreamcon Co., Ltd., Yangsan 50571, Gyeongnam, Korea; 5 Tyros Biotechnology Inc., Watertown, MA 02472, USA

**Keywords:** fluoroquinolones, ciprofloxacin, levofloxacin, bacterial keratitis, contact lens, drug-eluting lens, sustained drug release

## Abstract

The aim of this study is to prepare ciprofloxacin (CIP) or levofloxacin (LEVO)-incorporated and polydopamine (PDA)-coated nephrite composites for application in drug-eluting contact lenses. PDA was coated onto the surface of nephrite to improve antibacterial activity and to payload antibiotics. CIP or LEVO was incorporated into the PDA layer on the surface of nephrite. Furthermore, CIP-incorporated/PDA-coated nephrite composites were embedded into the contact lenses. PDA-coated nephrite composites showed dull and smooth surfaces according to the dopamine concentration while nephrite itself has sharp surface morphology. CIP- or LEVO-loaded/PDA-coated nephrite composites also have dull and smooth surface properties. Nano and/or sub-micron clusters were observed in field emission-scanning electron microscopy (FE-SEM) observation, indicating that PDA nanoparticles were accumulated and coated onto the surface of nephrite. Furthermore, CIP- or LEVO-incorporated/PDA-coated nephrite composites showed the sustained release of CIP or LEVO in vitro and these properties contributed to the enhanced antibacterial activity of composites compared to nephrite or PDA-coated nephrite composites. CIP-incorporated/PDA-coated nephrite composites were embedded in the contact lenses and then, in an antibacterial study, they showed higher bactericidal effect against *Staphylococcus aureus* (*S. aureus*) compared to nephrite itself or PDA-coated nephrite composites. We suggest that CIP- or LEVO-loaded/PDA-coated nephrite composite-embedded contact lenses are a promising candidate for therapeutic application.

## 1. Introduction

Extensive use of disposable contact lenses is regarded as one of the major causes of the spread of ulcerative keratitis [1,2,3]. In particular, the use of contact lenses is closely associated with the wide spread of microbial keratitis, which is a vision threatening disease and has become the source of severe complications in eye healthcare [4,5]. Microbial keratitis includes bacterial keratitis, fungal keratitis and Acanthamoeba keratitis [6]. Among them, bacterial keratitis causes rapid fulminant disease and is a viciousness factor of the permanent vision loss even though patients are treated clinically [7,8]. In bacterial keratitis, the ratio of Gram-positive bacteria is relatively higher than that of Gram-negative bacteria [9]. Among various treatment regimens, topical antibiotic treatment is the first-line treatment of bacterial keratitis [10,11]. However, the problem of drug resistance is significantly increased [11]. Goldstein et al. reported that *Staphylococcus aureus* (*S. aureus*) became resistant to ciprofloxacin, figures changing from 5.8% in 1993 to 35.0% in 1997, and to ofloxacin from 4.7% to 35.0% over the same period while Gram-negative bacteria showed relatively better susceptibility to theses antibiotics [11]. In clinical treatment, most of the patients have no improvement in the treatment of ciprofloxacin [12]. Thus, a novel treatment strategy is required for microbial keratitis.

Nephrite is a mineral tremolite or actinolite which is composed of calcium, magnesium and iron. In particular, we previously reported the antibacterial activity of nephrite against Gram-positive or Gram-negative bacteria [13,14,15]. We reported that the storage case for nephrite-containing contact lenses showed an inhibitory effect on the proliferation of *S. aureus* and thus nephrite can be used as a novel antibacterial material for keratitis [13]. They also showed antibacterial activity against *pseudomonas aeruginosa* (*P. aeruginosa*) [14]. Additionally, storage cases for nephrite-containing contact lenses showed an inhibitory effect against *Acanthamoeba lugdunensis* (*A. lugdunensis*) [15]. Therefore, the application of nephrite as an antibacterial material may help treat keratitis. However, nephrite itself is an inorganic material and it is not functional to payload or coat antibiotics. Thus, its antibacterial activity should be improved for application in contact lenses.

Coatings based on biocompatible polymers have been extensively investigated to improve biocompatibility and/or therapeutic properties of biomedical devices [16,17,18,19,20]. Biodegradable polymers such as methylmethacrylate/2-hydroxyethyl methacrylate copolymers, poly (ethylene oxide)/methacrylate copolymers and poly(lactic-co-glycolic acid copolymers) contributed to the sustained release of bioactive agents and the antimicrobial layer in the polymer coating efficiently prevented the infection of methicillin-resistant *S. aureus* [16,17,18]. Chitosan-based coating on the implant surfaces induces the inhibition of implant-associated infections and improves the durability/biocompatibility of implantable medical devices [19]. Otherwise, polydopamine (PDA), which is a nature-inspired polymer, has been widely studied for the coating of biomedical devices due to its biocompatibility, self-assembling property and universal adhesion onto the surface of various devices [20]. PDA is known to have antioxidant activity, i.e., PDA-crosslinked hydrogels efficiently scavenge 78.8% of free radicals in the biological system while only 1.4% of free radicals can be scavenged by hydrogels without PDA [21]. Furthermore, PDA has an antibacterial and antifungal efficacy against several microorganisms on the surface of medical devices by providing hydrophilic/charged surfaces [22,23,24]. Wang et al. reported that PDA-containing composites demonstrate excellent antibacterial and long-term antibacterial properties against Gram-negative *E. coli* and Gram-positive *S. aureus* [23]. Xiong et al. reported that PDA-functionalized nanofibers have superior antimicrobial/ROS scavenging activity and a beneficial effect on damaged tissue repair [24].

In this study, antibiotic-incorporated PDA was coated onto nephrites for application in drug-eluting contact lenses. Ciprofloxacin (CIP) or levofloxacin (LEVO) was used as model antibiotics. Antibiotics were incorporated into the PDA coating layer during PDA coating onto the surface of nephrite. Antibacterial efficacy of antibiotic-incorporated/PDA-coated nephrites was evaluated with *Staphylococcus aureus* (*S. aureus*) in vitro.

## 2. Materials and Methods

### 2.1. Materials

Nephrite was obtained from Sujenara Co. (Seoul, Korea). Dopamine, ciprofloxacin HCl (CIP), levofloxacin (LEVO), ethylene glycol dimethacrylate (EGDMA), azobisisobutyronitrile (AIBN), 2-hydroxyethyl methacrylate (2-HEMA) and tryptic soy broth (TSB) were purchased from Sigma-Aldrich Chem. Co. Ltd. (St. Louis, MO, USA). Dialysis membranes with 500~1000 g/mol and 8000 g/mol of molecular weight cut-off (MWCO) were purchased from Spectrum Lab., Inc. (New Brunswick, NJ, USA). All of the organic solvents, such as dichloromethane (DCM), dimethylsulfoxide (DMSO) and ethyl alcohol (EtOH), were used as an ultrapure grade.

### 2.2. Fabrication of PDA-Coated Nephrite and Antibiotic Incorporation

Preparation of the CIP (or LEVO)-incorporated/PDA-coated nephrite composite was depicted in Figure S1.

**Figure 1:**
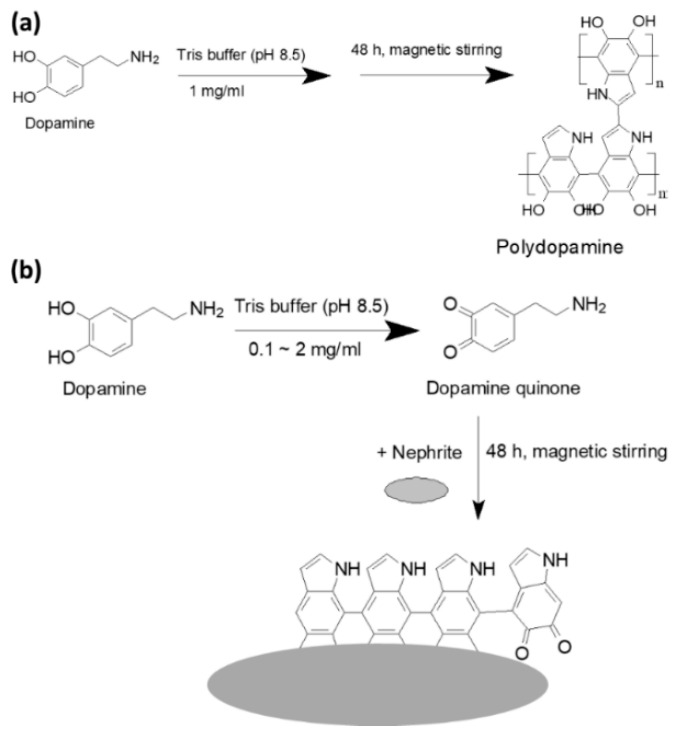
PDA copolymer synthesized by the polymerization of dopamine in the aqueous solution (**a**). PDA-coated nephrite composites by the polymerization of dopamine (**b**).

PDA nanoparticles (Figure 1): 1 g of dopamine was dissolved in 100 mL of Tris buffer (10 mM, pH 8.5) and then magnetically stirred for 48 h. This solution was changed to a black color during polymerization. Following this, PDA nanoparticles were harvested by centrifugation at 15,000 rpm for 30 min. Supernatants were discarded, centrifuged products were washed with distilled water and the products were harvested by centrifugation. The washing process was repeated three times to remove unreacted monomers and byproducts. This was reconstituted in deionized water and then the solids were recovered by lyophilization for 48 h.

PDA-coated nephrite composites: Dopamine (1 g) was dissolved in 100 mL Tris buffer (10 mM, pH 8.5) and then 1 g of nephrite powder was added. This solution was magnetically stirred for 48 h. After that, the solids were recovered by centrifugation at 9000 rpm for 10 min. Supernatants were discarded, centrifuged products were washed with water and then the products were harvested by centrifugation. The washing process was repeated three times to remove unreacted monomers and byproducts. This was reconstituted in deionized water and then the solids were recovered by lyophilization for 48 h.

CIP (or LEVO)-incorporated/PDA-coated nephrite composites: 0.2 g of CIP (or LEVO) and 1 g of dopamine were dissolved in 100 mL Tris buffer (10 mM, pH 8.5). After that, 1 g of nephrite powder was added. This solution was magnetically stirred for 48 h. After that, solids were recovered by centrifugation at 9000 rpm for 10 min. Supernatants were discarded and centrifuged products were washed with water and then harvested by centrifugation. The washing process was repeated three times to remove unreacted monomers and byproducts. This was reconstituted in deionized water and then the solids were recovered by lyophilization for 48 h.

For the contact lenses embedded with CIP-incorporated/PDA-coated nephrite composites, 2-HEMA, EGDMA and AIBN were poured into pre-designed corrosion disks and were then printed in the shape of a lens. The composition of the soft contact lenses is demonstrated in Table 1. This was attached to silicon pad. Following this, the silicon pad was printed into the upper mold and then polymerized by UV-irradiation for 60 min until 90~95% polymerization degree. Following this, the polymer was spread out onto the lower mold and then introduced into the upper mold along with CIP-incorporated/PDA-coated nephrite composite. Then, these were reacted to form contact lenses.

### 2.3. Morphology of CIP (or LEVO)-Incorporated/PDA-Coated Nephrite Composites

Composite morphology was observed with a field-emission scanning electron microscope (S-4800; Hitachi, Tokyo, Japan). Observation of each compound was carried out at 20 °C and 25 kV. For the observation of nephrite solids, intact nephrite powder was used. Lyophilized solids such as PDA nanoparticles, PDA-coated nephrite and CIP-incorporated/PDA-coated nephrite composites were placed onto copper grid using double-sided tape to observe the surface morphologies. The samples were coated with gold/palladium using an ion sputter (Jeol Fine Coat Ion Sputter, JFC-1100, JEOL, Ltd., Tokyo, Japan).

### 2.4. Crystalline Properties of CIP-Incorporated/PDA-Coated Nephrite Composites

Lyophilized solids such as nephrite, PDA nanoparticles, PDA-coated nephrite, CIP-incorporated/PDA-coated nephrite composites and CIP were used to measure the crystalline properties using XRD diffractometers. Rigaku D/Max-1200 (Rigaku, Tokyo, Japan) equipped with Ni-filtered Cu Kα radiation (40 kV, 20 mA) was used to analyze XRD diffractogram of the composites. All measurements were carried out at room temperature. The conditions of the powder XRD measurement was as follows:

- Data type = binary; goniometer = 1; attachment = 1; scan mode = continuous.
- Mode 2 (R/T) = reflection; scan axis = 2*θ*/*θ*.
- Start angle = 10.000; stop angle = 80.000; scan speed = 5.000; sampling interval = 0.050; *θ* angle = 5.000; 2*θ* angle = 10.000; fixed time = 0.01; full scale = 1000; counting unit = CPS; target= Cu.
- Wavelength Ka1 = 1.540510; wavelength Ka2 = 1.544330; wavelength Ka = 1.541780; wavelength Kb = 1.392170; 40.0 kV; 20.0 mA.

### 2.5. Drug Content Measurement and Drug Release Study

CIP contents in the composites: 20 mg lyophilized solids of CIP (or LEVO)-incorporated/PDA-coated nephrite composites were distributed in 5 mL acetone for CIP or 5 mL chloroform for LEVO. This solution was magnetically stirred for 12 h. In total, 2 mL of this solution was centrifuged at 15,000 rpm for 30 min. Then, supernatants were used to measure CIP concentration at 277 nm and LEVO concentration at 288 nm using UV-VIS spectrophotometer (UV-VIS spectrophotometer 1601, Shimadzu Co. Tokyo, Japan). CIP (or LEVO) contents = (CIP (or LEVO) weight/total composite weight) × 100. For comparison, a PDA-coated nephrite composite was also treated similarly as described above and then the absorption value was compensated.

CIP or LEVO release from composite: Composite (10 mg) was reconstituted in 20 mL of phosphate-buffered saline (PBS, pH 7.4, 0.01 M). This was put into 50 mL falcon tube and then agitated at 100 rpm (37 °C). In total, 2 mL of this solution was taken and then centrifuged at 15,000 rpm for 30 min. Then, CIP concentration, which is released from composites, was measured with a UV-spectrophotometer (UV-1601, Shimadzu Co. Ltd., Kyoto, Japan) at 277 nm for CIP and 288 nm for LEVO. For comparison, a PDA-coated nephrite composite was also treated similarly as described above and then the absorption value was compensated. For comparison with free CIP liberation, CIP (or LEVO) dissolved in deionized water was introduced in dialysis membrane (MWCO: 500~1000 g/mol) and then similarly performed drug release study.

### 2.6. Antibacterial Activity In Vitro

*S. aureus* was obtained from the Korean Collection for Oral Microbiology (Gwangju, Korea). For the evaluation of the antibacterial activity of the composites, stock solutions were prepared and dilutions were made according to the Clinical Laboratory Standards Institute-CLSI (formerly NCCLS) M7-A6 method (15 National Committee for Clinical Laboratory Standards 2003). *S. aureus* were provided by Korea Collection for Type Cultures (KCTC, Jeongeup-si, Korea). Frozen stocks were cultured in TSB media (plus 10% NaCl) at 37 °C and then this was sub-cultured. Following this, CIP or composites were added to the bacterial solutions. One day later, the growth of the bacteria was evaluated by optical density at 600 nm (UV-spectrophotometer 1201, Shimadzu Co. Ltd., Kyoto, Japan). All experiments were carried out in triplicate and expressed as average ± standard deviation (S.D.).

To evaluate the sustained released properties of the composites, CIP-incorporated/PDA-coated nephrite composites in PBS solution (as a CIP concentration, 0.1 mg CIP/0.5 mL PBS) were introduced into a dialysis membrane (MWCO: 8000 g/mol). Free CIP was also dissolved in PBS (0.1 mg/0.5 mL) and was put into the dialysis membrane. Same quantity of PDA-coated nephrite composites was also put into the dialysis membrane to compare. PBS solution (0.5 mL) was put into the dialysis membrane for control treatment. Each of these were introduced into 10 mL of *S. aureus* (1 × 10^6^/mL, TSB media (plus 10% NaCl)). To imitate the in vivo environment, 5 mL of culture media was exchanged with fresh media at every 30 min intervals for 2 h, 1 h intervals for 6 h and then 2 h intervals until 44 h. After 48 h of incubation, the growth of the bacteria was estimated with optical density at 600 nm.

### 2.7. Cell Cytotoxicity

For cell cytotoxicity evaluation of CIP and CIP- or LEVO-incorporated/PDA-coated nephrite composites, ARPE-19 human retinal pigmented epithelial cells were obtained from American Type Culture Collection (ATCC, Manassas, VA, USA). ARPE-19 cells were maintained with Dulbecco’s Modified Eagle Medium/F-12 (DMEM/F12, Gibco^®^, Grand Island, NY, USA) media supplemented with 10% heat-inactivated fetal bovine serum (FBS) (Gibco^®^, Life Technologies Co., Carlsbad, CA, USA) and 1% penicillin/streptomycin. For cell viability, ARPE-19 cells (2 × 10^4^ cells/well) seeded in 96-well plates were cultured overnight in 5% CO2 at 37 °C and then treated with CIP, LEVO and CIP- or LEVO-incorporated/PDA-coated nephrite composites. Free CIP, free LEVO and CIP- or LEVO-incorporated/PDA-coated nephrite composites were dissolved or reconstituted in deionized water and then diluted with cell culture media. Following this, cells were treated with free CIP, free LEVO and CIP- or LEVO-incorporated/PDA-coated nephrite composites. Then, 24 h later, 30 μL of MTT reagent (5 mg/mL in PBS) was added to the cells and then incubated for 4 h at 37 °C. Following this, supernatants were discarded and replaced with 100 µL of DMSO. The cell viability was measured at 570 nm (Infinite M200 pro microplate reader, Tecan, Mannedorf, Switzerland).

### 2.8. Statistical Analysis

Statistical analysis of the results was evaluated with the Student’s *t*-test and *p* values lower than 0.01 were considered as a significant value.

## 3. Results

### 3.1. CIP-Incorporated and PDA-Coated Nephrite Composites

**Figure 2:**
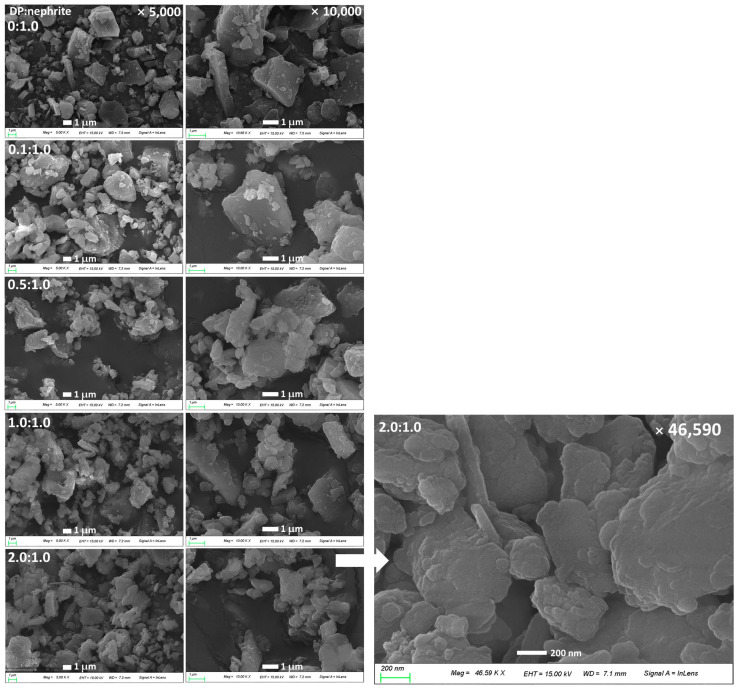
Morphological observation of nephrite and PDA-coated nephrite composites.

Figure 1 and Figure 2 shows the PDA coating onto the surface of nephrite. Prior to PDA coating onto the nephrite surface, polymerization and then nanoparticle formation were studied as shown in Figure 1a and Figure S2. Dopamine can be polymerized to polydopamine in an aqueous solution. As shown in Figure S2, tiny PDA nanoparticles less than 50 nm were formed by the polymerization of dopamine. This result indicated that nanoparticles can be formed by the polymerization of dopamine and then this can be used as a bio-functional material. Figure 1b shows the PDA-coating process onto the surface of nephrite using dopamine monomer. Furthermore, various ratios of dopamine vs. nephrite were used to coat nephrite and then their morphologies were investigated as shown in Figure 2. As shown in Figure 2, nephrite itself has irregular and sharp surfaces. When dopamine was added to be polymerized onto the surface of nephrite, surface morphology of nephrite became smooth due to the increase in the dopamine ratio. Especially, small particles were observed in the higher dopamine ratio of the PDA-coated nephrite composite as shown in Figure 2. These results indicated that PDA particles might be formed on the surface of nephrite.

For the incorporation of antibiotics in the composites, CIP or LEVO was introduced to the fabrication process of PDA-coated nephrite composites as summarized in Table 2. As shown in Table 2, loading efficiency of CIP (experimental CIP contents) was increased when the dopamine ratio was relatively increased. Lower ratio of dopamine induced a significant loss of CIP. These results are due to the fact that the polymerization of dopamine to the PDA on the nephrite surfaces might be higher in the higher feeding ratio of dopamine and then CIP can be easily incorporated into the PDA layer compared to the lower dopamine ratio. When the CIP feeding ratio was decreased to 0.1 (CIP/dopamine/nephrite), experimental drug contents were 3.7%, *w*/*w*. In the case of LEVO-incorporated composites, experimental LEVO contents were 7.4%, *w*/*w*.

**Figure 3:**
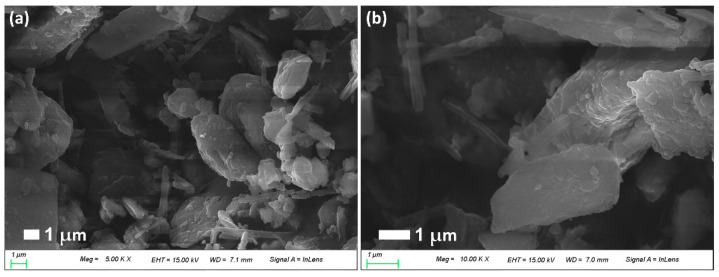
Antibiotics-incorporated/PDA-coated nephrite composites: (**a**) CIP-incorporated (CIP/dopamine/nephrite ratio = 0.2/1.0/1.0); (**b**) LEVO-incorporated (LEVO/dopamine/nephrite ratio = 0.2/1.0/1.0).

Furthermore, CIP-incorporated/PDA-coated nephrite composites also showed dull and smooth surfaces as shown in Figure 3a,b. Furthermore, nano and/or sub-micron clusters were observed in the higher concentration of dopamine, indicating that PDA particles must be accumulated on the surface of nephrite and its density was increased according to the increased ratio of dopamine as shown in Figure 2. These results indicated that dopamine was polymerized on the surface of nephrite and then modified the surface characteristics of nephrite.

**Figure 4:**
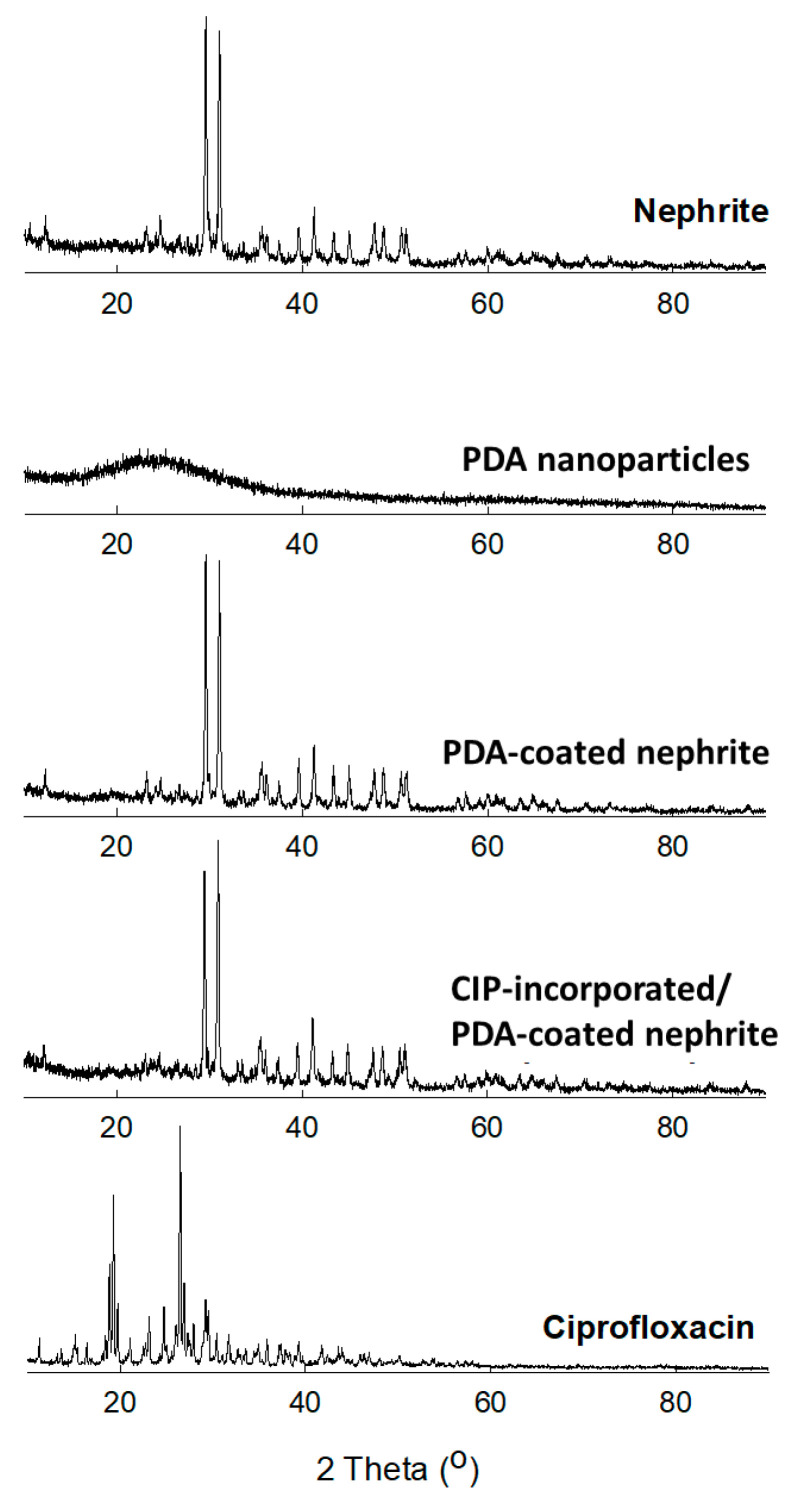
Powder XRD diffractograms of nephrite, PDA nanoparticles, PDA-coated nephrite composites (Dopamine/nephrite ratio = 1.0/1.0), CIP-incorporated/PDA-coated nephrite composites (CIP/dopamine/nephrite ratio = 0.2/1.0/1.0) and ciprofloxacin HCl (Ciprofloxacin).

Figure 4 shows the powder XRD diffraction of nephrite, PDA-coated nephrite composites and CIP-incorporated/PDA-coated nephrite composites. As shown in Figure 4, nephrite itself caused sharp crystalline peaks while PDA nanoparticles caused smooth and non-crystalline peak characteristics. Otherwise, PDA-coated nephrite composites caused almost similar peak characteristics compared to nephrite. When CIP was incorporated, CIP-incorporated/PDA-coated nephrite composites also produced similar peak characteristics compared to nephrite itself, indicating that CIP was incorporated into the PDA layer on the surface of nephrite. Furthermore, these results indicated that PDA coating and/or CIP incorporation did not change intrinsic crystalline properties of nephrite.

### 3.2. Drug Release Study

**Figure 5:**
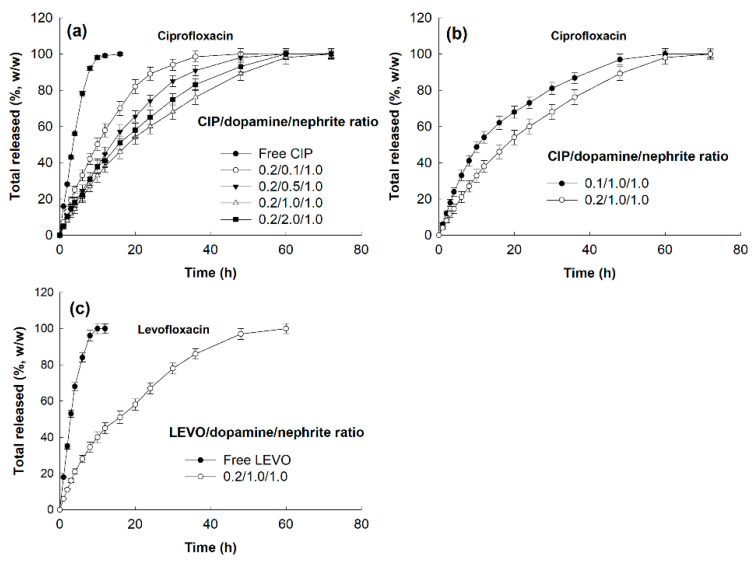
Comparison of pure drug liberation (Free CIP) and CIP (or LEVO) release from CIP (or LEVO)-incorporated/PDA-coated nephrite composites. (**a**) The effect of dopamine ratio on the CIP release from CIP-incorporated/PDA-coated nephrite composites. (**b**) The effect of CIP weight ratio on the CIP release from CIP-incorporated/PDA-coated nephrite composites. (**c**) LEVO release from LEVO-incorporated/PDA-coated nephrite composites. LEVO/PDA/Nephrite: LEVO-incorporated/PDA-coated nephrite composites.

CIP and LEVO release study was performed in vitro as shown in Figure 5. As shown in Figure 5a–c, CIP was continuously released from antibiotics-incorporated/PDA-coated nephrite composites over 2 days while free CIP was rapidly liberated from the dialysis tube. When the dopamine ratio in the composites was increased, the drug release rate was relatively delayed until a dopamine ratio of 1.0 (CIP/dopamine/nephrite ratio = 0.2/1.0/1.0), as shown in Figure 5a. However, the drug release rate at the highest dopamine ratio of 2.0 (CIP/dopamine/nephrite ratio = 0.2/2.0/1.0) was relatively faster than the dopamine ratio of 1.0. These results might be due to the fact that PDA nanoparticles can be preferentially formed at higher dopamine ratios, as shown in Figure S2.

Furthermore, LEVO was also released from LEVO-incorporated/PDA-coated nephrite composites over 2 days with a sustained release manner while free LEVO was rapidly liberated from the dialysis tube. These results indicated that CIP (or LEVO)-incorporated/PDA-coated nephrite composites have sustained drug release properties in an aqueous solution. Furthermore, sustained release properties of CIP- or LEVO-incorporated/PDA-coated nephrite composites may provide continuous antibacterial activity while antibiotic solution only provides temporary antibiotic activity.

### 3.3. Antibacterial Activity

**Figure 6:**
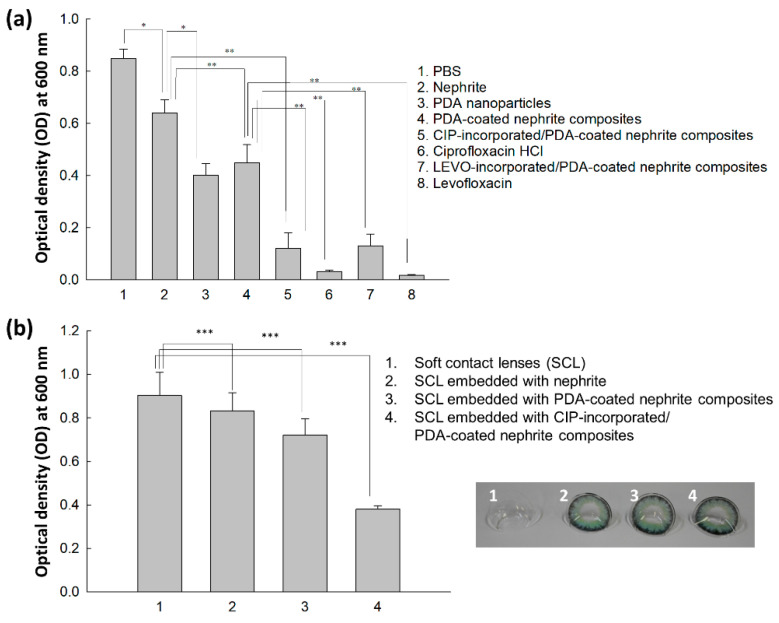
Antibacterial activity of CIP-incorporated/PDA-coated nephrite composites (CIP/dopamine/nephrite ratio = 0.2/1.0/1.0) against S. aureus in vitro (**a**). CIP or LEVO dosage was adjusted to 5 µg/mL. Dosage of PDA nanoparticles was 50 µg PDA/mL. Dosage of PDA-coated nephrite composites was 100 µg/mL. *, **: *p* < 0.01. Antibacterial activity of soft contact lenses (SCL) embedded with CIP-incorporated/PDA-coated nephrite composites (CIP/dopamine/nephrite ratio = 0.2/1.0/1.0) against *S. aureus* in vitro (**b**). ***: *p* < 0.01.

Antibacterial activity of nephrite, antibiotics, PDA nanoparticles, PDA-coated nephrite composites, CIP (or LEVO)-incorporated/PDA-coated nephrite composites and soft contact lenses embedded with CIP-incorporated/PDA-coated nephrite composites was evaluated with *S. aureus* for 1 day (Figure 6a). As shown in Figure 6a, nephrite itself has limited antibacterial activity against *S. aureus* while PDA nanoparticles or PDA-coated nephrite composites showed higher antibacterial activity. In particular, CIP-incorporated/PDA-coated nephrite composites and LEVO-incorporated/PDA-coated nephrite composites showed significantly higher antibacterial activity compared to nephrite, PDA nanoparticles or PDA-coated nephrite composites. These results indicated that CIP-incorporated and/or LEVO-incorporated/PDA-coated nephrite composites have reasonable antibacterial activity against *S. aureus*. In Figure 6a, free CIP or free LEVO showed higher antibacterial activity than those of CIP-incorporated and/or LEVO-incorporated/PDA-coated nephrite composites. These results might be due to the fact that CIP-incorporated and/or LEVO-incorporated/PDA-coated nephrite composites have sustained drug release properties; thus the practical concentration of released CIP or LEVO must be lower than the free drug. Figure 6b shows the antibacterial activity of CIP-incorporated/PDA-coated nephrite composite-embedded soft contact lenses (SCL) against *S. aureus*. As shown in Figure 6b, SCL embedded with nephrite and PDA-coated nephrite composites showed little gain in inhibitory effect against the growth of bacteria compared to SCL itself. Furthermore, SCL embedded with CIP-incorporated and LEVO-incorporated/PDA-coated nephrite composites showed significantly higher inhibitory effect against the growth of S. aureus. These results indicated that SCL embedded with CIP-incorporated or LEVO-incorporated/PDA-coated nephrite composites can be used as a promising candidate to inhibit bacterial infection.

**Figure 7:**
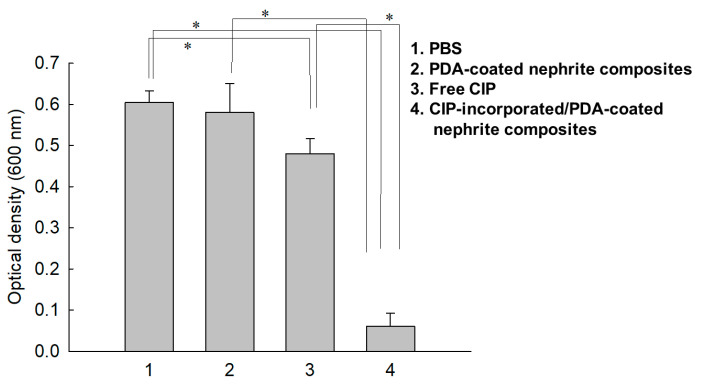
Antibacterial activity of CIP-incorporated/PDA-coated nephrite composites (CIP/dopamine/nephrite ratio = 0.2/1.0/1.0). For CIP and composite treatment, CIP concentration was adjusted to (0.1 mg CIP/0.5 mL PBS) and then each component was put into a dialysis membrane. PBS was used as a control and the same amount of PDA-coated nephrite composites was used for comparison. *: *p* < 0.01.

To evaluate the sustained release properties of CIP-incorporated/PDA-coated nephrite composites against bacterial growth, they were introduced into a dialysis membrane and then media were exchanged to mimic an in vivo environment as shown in Figure 7. As shown in Figure 7, PBS or PDA-coated nephrite composites did not practically affect the growth of bacteria while CIP-incorporated/PDA-coated nephrite composites significantly inhibited bacterial growth. Even though free-CIP treatment also inhibited bacterial growth initially, the extent of bacterial growth was five times higher than the treatment of CIP-incorporated/PDA-coated nephrite composites. These results were due to the sustained drug release properties of CIP- or LEVO-incorporated/PDA-coated nephrite composites; thus, they maintained antibacterial activity during the fabrication process or drug release period.

**Figure 8:**
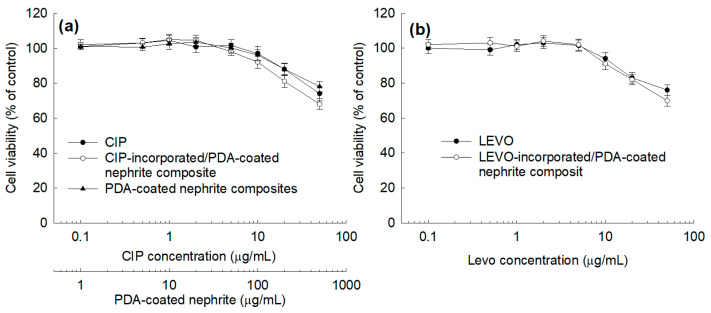
Cell cytotoxicity of CIP- or LEVO-incorporated/PDA-coated nephrite composites (CIP (or LEVO)/dopamine/nephrite ratio = 0.2/1.0/1.0) against ARPE-19 human retinal pigmented epithelial cells. (**a**) The effect of free CIP, CIP-incorporated/PDA-coated nephrite composites and PDA-coated nephrite composites; (**b**) The effect of free LEVO or LEVO-incorporated/PDA-coated nephrite composites. Overall, 2 × 10^4^ ARPE-19 cells in 96-well plates were treated with each compound for 24 h. Cell viability was evaluated with an MTT assay.

To evaluate the biocompatibility of CIP- or LEVO-incorporated/PDA-coated nephrite composites, they were cultured with ARPE-19 cells in vitro as shown in Figure 8. When the cells were treated with CIP-incorporated/PDA-coated nephrite composites (Figure 8a), the viability of the ARPE-19 cells was higher than 80% until 20 μg/mL of CIP concentration as well as free CIP. Furthermore, the viability of ARPE-19 cells was also maintained higher than 80% until 20 μg/mL of LEVO concentration both for free LEVO and LEVO-incorporated/PDA-coated nephrite composites (Figure 8b). Furthermore, PDA-coated nephrite composites also maintained higher than 80% cell viability at the same treatment dosage compared to CIP- or LEVO-incorporated/PDA-coated nephrite composites. These results indicated that CIP- or LEVO-incorporated/PDA-coated nephrite composites have low cytotoxicity against ARPE-19 cells and therefore they have no acute toxicity against normal cells of the ocular compartment.

## 4. Discussion

Since nephrite itself has antibacterial activity against Gram-negative or Gram-positive bacteria, nephrite was selected as an antibacterial material for SCL [13,14,15]. Additionally, PDA was coated onto the surface of nephrite to emphasize antibacterial activity because it is frequently employed as an antibacterial coating material for various implants [22]. PDA coating on the surface of a biomaterial or implant endowed bioactive surfaces for therapeutic purposes [24,25,26]. Furthermore, a PDA layer on the biofilm endowed a synergistic effect to kill bacteria and a self-cleaning process [26]. In our results, the surface of nephrite was successfully coated with polydopamine, which modified the surface from sharp to dull surfaces according to dopamine concentration. As shown in Figure 2, PDA formed as small particles on the surface of nephrite. Murari et al. also reported that PDA forms nano or submicron clusters on the poly(ε-caprolactone) (PCL) substrates and then surface morphology was changed according to the polymerization time [25]. In our results, it is likely that nano and/or sub-micron clusters were formed by PDA coating as shown in Figure 2. These might be due to the PDA particles attached onto the surface of nephrite, which then modified the surface of nephrite. Furthermore, PDA coating on the surface of nephrite endows a bactericidal effect to the composite, i.e., PDA-coated nephrite composites revealed increased inhibitory effect against the growth of S. aureus compared to nephrite itself as shown in Figure 6. PDA nanoparticles themselves also have a bactericidal effect. Furthermore, the PDA layer on the surface of nephrite acts as a platform for drug payload. That is, CIP or LEVO was loaded into the PDA layer of nephrite composites and then they were continuously released from the composite as shown in Figure 5. Furthermore, CIP and/or LEVO-incorporated/PDA-coated nephrite composites showed improved antibacterial activity as shown in Figure 6a. Even though intact CIP or LEVO showed higher antibacterial activity against *S. aureus* as shown in Figure 6a, topical antimicrobial therapy using CIP has limited clinical improvement, i.e., less than 20% of patients responded to topical treatment in clinical studies against Staphylococcus keratitis patients [12]. However, antibacterial activity of CIP and/or LEVO-incorporated/PDA-coated nephrite composites must be maintained longer during an in vivo environment. In the case of pure nephrite, it only has little antibacterial activity when it embedded in the soft contact lenses (SCL) as shown in Figure 6b. Even though there are little differences in antibacterial activity, nephrite-embedded lenses (SCL embedded with nephrite) and PDA-coated nephrite composite-embedded lenses (SCL embedded with PDA-coated nephrite composites) showed gradual increase in antibacterial activity as shown in Figure 6b. However, SCL embedded with CIP-incorporated PDA-coated nephrite composites showed distinct antibacterial activity in vitro. These results indicated that drug-eluting SCL has the potential to prevent ulcerative keratitis. In a previous study, sustained release properties of antibiotic-encapsulated poly(DL-lactide-co-glycolide) nanoparticles effectively inhibited bacterial growth in an in vivo experiment while they showed lower antibacterial activity than that of intact antibiotics in vitro [27]. Practically, we also obtained similar results in an in vitro study for the evaluation of antibacterial activity of CIP-incorporated/PDA-coated nephrite composites, i.e., bacteria growth was significantly inhibited when using CIP-incorporated/PDA-coated nephrite composites as shown in Figure 7. However, the treatment of free CIP was not significantly changed compared to those of PBS or PDA-coated nephrite composites. These results indicated that the sustained release properties of composites contribute to the continuous antibacterial activity. In particular, free CIP treatment in Figure 7 is quite similar to the environment of topical CIP treatment because CIP must be rapidly removed from the body in the case of topical CIP treatment. Gao et al. also reported that CIP-incorporated PDA/glycol chitosan nanoparticles have sustained release properties awhich efficiently inhibit the growth of S. aureus [28]. They argued that CIP-incorporated nanoparticles have bactericidal effect against various bacteria in an in vitro and animal infection model using *S. aureus*. Furthermore, CIP- or LEVO-incorporated/PDA-coated nephrite composites have low cytotoxicity against normal human retinal pigmented epithelial cells as shown in Figure 8. The viability of ARPE-19 cells was higher than 80% until 20 μg/mL CIP or LEVO for both free antibiotics and their composites.

Drug-eluting contact lenses have been extensively investigated for the diagnosis and therapeutic purposes of retinal disease [29,30,31,32,33]. For example, many researchers investigated the bioinspired polymeric systems for the improvement of biocompatibility, specific molecular recognition, stimuli-responsive system for drug delivery and diagnosis of systemic disease [29,30,31]. Gade et al. reported that polymeric contact lenses prepared from chitosan, glycerol and poly(ethylene glycol) (PEG) payload moxifloxacin and dexamethasone, and then showed sustained drug release properties [32]. They argued that drug-eluting contact lenses showed significantly higher corneal drug distribution compared to topical drug solutions. Furthermore, Bengani et al. also reported that dexamethasone-releasing contact lenses delivered drugs over an extended period of time and then efficiently inhibited neovascularization or inflammation for 7 days [33]. In our results, contact lenses embedded with CIP-incorporated/PDA-coated nephrite composites showed increased antibacterial activity against *S. aureus* compared contact lenses themselves, nephrite-embedded contact lenses or PDA-coated nephrite-embedded contact lenses as shown in Figure 7. These results may support in the suppression of intraocular infection.

## 5. Conclusions

The aim of this study was to prepare CIP-incorporated and PDA-coated nephrite composites for application in drug-eluting contact lenses. PDA was coated onto the surface of nephrite to improve antibacterial activity and to payload antibiotics. PDA-coated nephrite composites showed dull and smooth surfaces according to the dopamine concentration while nephrite itself has sharp surface morphology. CIP or LEVO-loaded/PDA-coated nephrite composites also have dull and smooth surface properties. Nano and/or sub-micron clusters were observed in FE-SEM observation, indicating that PDA particles were accumulated and coated onto the surface of nephrite. Furthermore, CIP- or LEVO-incorporated/PDA-coated nephrite composites showed the sustained release behavior of CIP or LEVO in vitro and then these properties may contribute to the enhanced antibacterial activity of composites compared to pure nephrite or PDA-coated nephrite composites. CIP-incorporated/PDA-coated nephrite composites were embedded in the SCL and then, in an antibacterial study, they showed an increased bactericidal effect against *S. aureus*. We suggest that SCL embedded with CIP or LEVO-loaded/PDA-coated nephrite composites is a promising candidate for therapeutic application.

## Figures and Tables

**Table 1:** Composition and content of soft contact lenses.

Composition	HEMA Soft Contact Lenses (%, *w*/*w*)
2-HEMA	98.9 1.0 0.1
EGDMA AIBN	

**Table 2:** Characterization of CIP (or LEVO)-incorporated/PDA-coated nephrite composites.

Composition Ratio (g) Antibiotics/Dopamine/Nephrite	Drug Contents (%, *w*/*w*)	
	Theoretical ^a^	Experimental ^b^
PDA-coated nephrite composites *		
0/1.0/1.0	-	-
CIP-incorporated/ PDA-coated		
nephrite composites		
0.2/0.1/1.0	15.4	
0.2/0.5/1.0	11.8	6.8
0.2/1.0/1.0	9.1	7.4
0.2/2.0/1.0	6.3	3.1
0.1/1.0/1.0	4.8	3.7
LEVOP-incorporated/ PDA-coated		
nephrite composites		
0.2/1.0/1.0	9.1	7.4

^a^ Theoretical drug contents (%, *w*/*w*) = (Feed weight of antibiotics/Total weight of (antibiotics + dopamine + nephrite)) × 100. ^b^ Experimental drug contents (%, *w*/*w*) = (Antibiotic weight in composites/Total weight of composite) × 100. * For PDA-coated nephrite composites, antibiotics were not provided. When UV-VIS spectra were measured to determine drug contents, PDA-coated nephrite composites were used as a blank.

## Data Availability

Not applicable.

## Supplementary Materials

The following supporting information can be downloaded at: https://www.mdpi.com/article/10.3390/ma15144823/s1, Figure S1: Process of PDA nanoparticles (a), PDA-coated nephrite composite (b), CIP-incorporated/PDA-coated nephrite composite (c) and the resultant powder of each composite (d); Figure S2: FE-SEM image of PDA nanoparticles.
