# Supplementary material for: Antibacterial Activity of Antibiotic-Releasing Polydopamine-Coated Nephrite Composites for Application in Drug-Eluting Contact Lens
Source: Materials (Basel). 2022 Jul 11;15(14):4823. doi: 10.3390/ma15144823 (PMC9315558; doi:10.3390/ma15144823)
Supplement: Supplementary file 1 [file materials-15-04823-s001.zip › materials-1719426-supplementary.pdf]

Supplementary materials

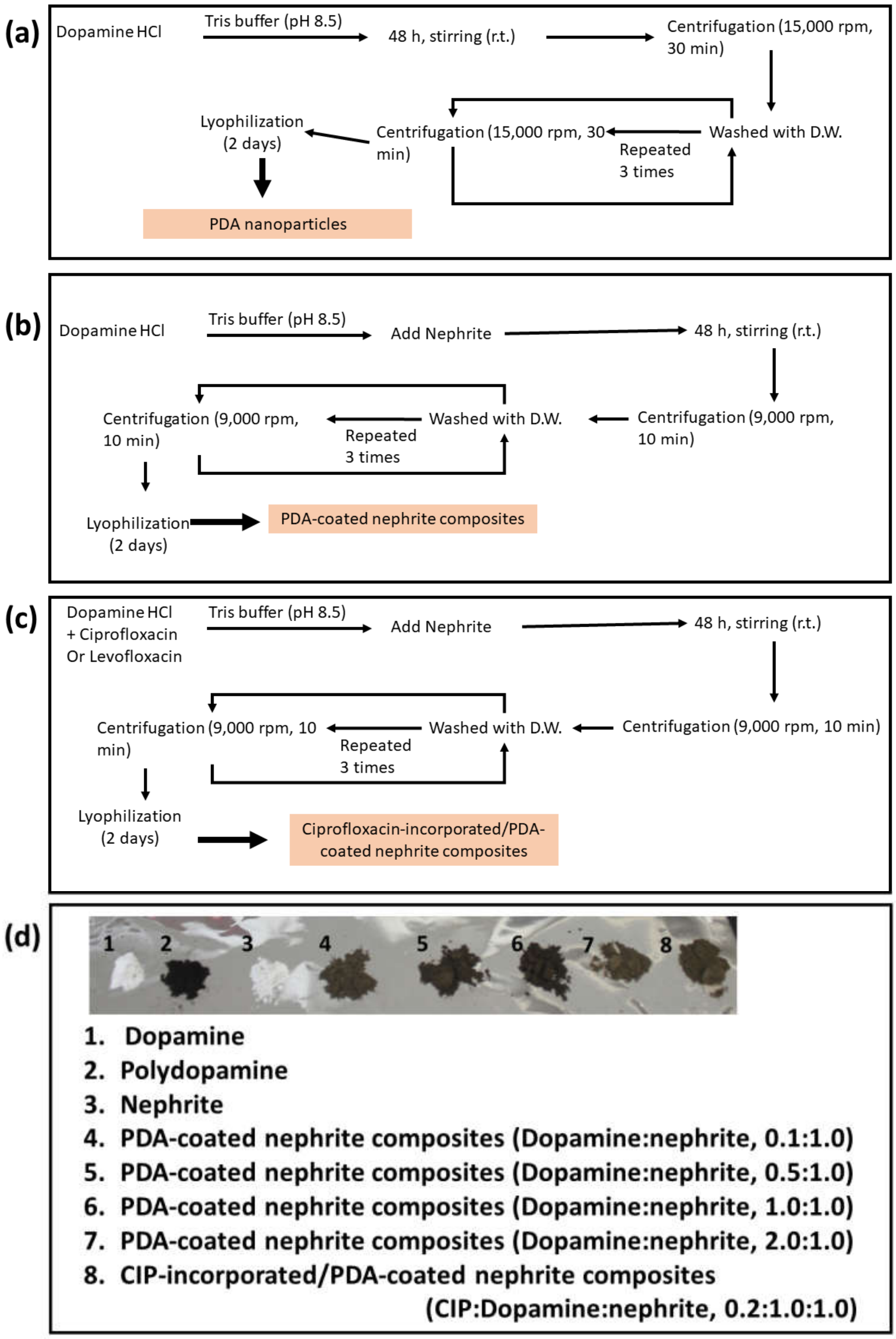

Figure S1. Process of PDA nanoparticles (a), PDA-coated nephrite composite (b), CIP-incorporated/PDA-coated nephrite composite (c) and the resultant powder of each composite (d).

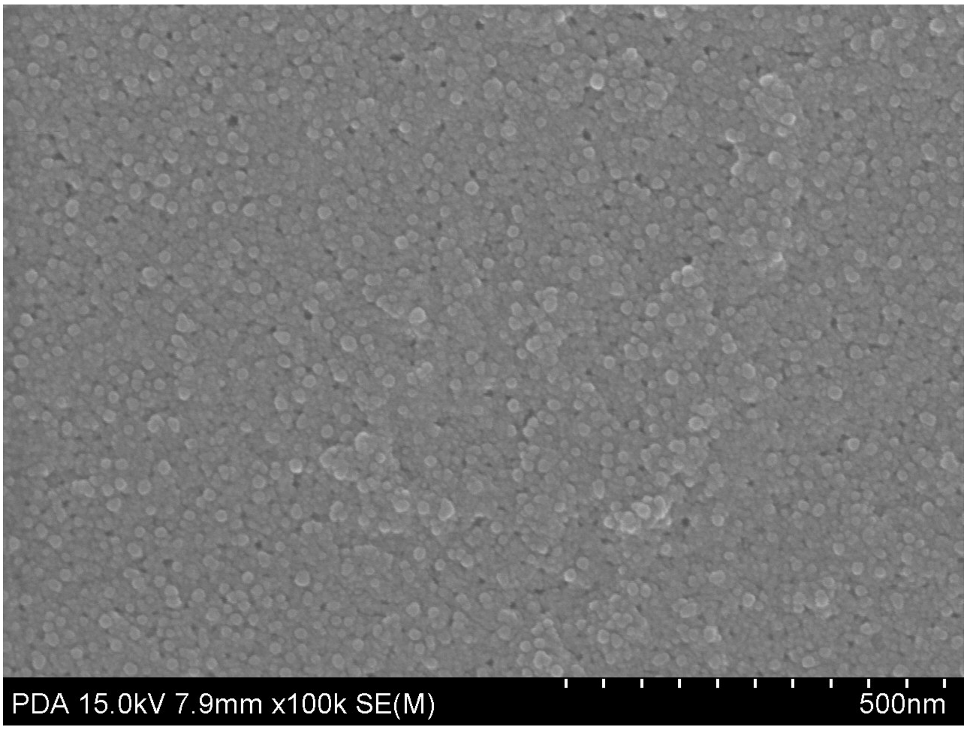

Figure S2. FE-SEM image of PDA nanoparticles.
